# Modeling Tdp‐43 dependent misprocessing of Stmn2 in vivo

**DOI:** 10.1002/alz70855_101194

**Published:** 2025-12-23

**Authors:** Sarah R Pickles, Luca Gaiani, Lilian Lin, Karen Jansen‐West, Jimei Tong, Giulia Del Rosso, Lillian Daughrity, Yongjie Zhang, Leonard Petrucelli

**Affiliations:** ^1^ Mayo Clinic, Jacksonville, FL, USA

## Abstract

**Background:**

Some cases of Alzheimer's Disease (AD) and Frontotemporal Dementia (FTD) feature the mislocalization of the normally nuclear TAR DNA binding protein (TDP‐43) to the cytoplasm where it is incorporated into inclusions in affected brain regions. TDP‐43 plays a crucial role in the suppression of cryptic exons in the mature RNA of many targets in both AD and FTD. One such target, Stathmin‐2 (STMN2), is misspliced in the absence of TDP‐43 leading to a reduction of STMN2 RNA and protein while the levels of the cryptic splice variant is increased. Cryptic exons are not conserved across species, meaning previous Tdp‐43 models did not capture *Stmn2* processing defects and Stmn2 loss of function (LOF). We hypothesize that STMN2 LOF contributes to cognitive phenotypes observed in AD and FTD.

**Method:**

We have generated a novel mouse that more accurately models human disease mechanisms by expressing short‐hairpin (shRNA) via adeno associated virus in the central nervous system of a recently described humanized *Stmn2* mouse model in which *Stmn2* splicing is dependent on Tdp‐43. In this mouse we will simultaneously model Tdp‐43 loss of function and aberrant *Stmn2* splicing.

**Result:**

Our preliminary experiments demonstrate that humanized Stmn2 mice expressing shRNA against *Tardbp* have a reduction of *Tardbp* RNA and TDP‐43 protein compared to mice expressing a non‐targeting shRNA. Tdp‐43 knock‐down is accompanied by cryptic splicing of endogenous mouse targets, *Sortilin 1* and *Translin*. As anticipated, we also detected cryptic *Stmn2* splicing, suggesting that correct *Stmn2* splicing is dependent on Tdp‐43 in this model. We will further characterize motor and cognitive functions in addition to neurodegeneration and gliosis in these animals.

**Conclusion:**

We anticipate this novel model, and our subsequent studies will identify relevant disease mechanisms and provide an invaluable tool with which to investigate therapies for AD and FTD.

